# circIFT80 Functions as a ceRNA for miR-142, miR-568, and miR-634 and Promotes the Progression of Colorectal Cancer by Targeting *β*-Catenin

**DOI:** 10.1155/2022/8081246

**Published:** 2022-06-23

**Authors:** Ning Liu, Fan Jiang, Zhiju Chen, Xin Liu, F. U. Zhiming, Bao-chun Wang, Yunfu LV

**Affiliations:** ^1^Department of Gastrointestinal Surgery, Hainan General Hospital, Hainan Affiliated Hospital of Hainan Medical University, Haikou, Hainan 570311, China; ^2^Department of the Center of Gerontology, Hainan General Hospital, Hainan Affiliated Hospital of Hainan Medical University, Haikou, Hainan 570311, China; ^3^Department of General Surgery, Hainan General Hospital, Hainan Affiliated Hospital of Hainan Medical University, Haikou, Hainan 570311, China

## Abstract

Colorectal cancer (CRC) is the third most common form of malignant tumor and is characterized by high rates of proliferation and metastases. Circular RNAs (circRNAs) are a form of noncoding and closed loop RNA molecules and play vital roles in the progression of various types of cancer in humans. Here, we used circRNA microarray sequencing technology to analyze the different circRNAs between CRC tissues and normal tissues and explore the role of circIFT80 in progression of colorectal cancer. In this present study, we found that circIFT80 was abnormally overexpression in colorectal cancer tissues and tumor cells. While knockout circIFT80 in HT29 cell or SW480 cells, the proliferation, and migration of the cells were inhibited, the cell cycle was arrested in G2/M phase, and the cell apoptosis was increased. And then, we found circIFT80-positive correlation with CTNNB1 (*β*-catenin) by sponging miR-142, miR-568, and miR-634 upregulated the gene expression. These miRNAs which targeted *β*-catenin mRNA were confirmed by dual-luciferase reporter system and RNA-pulldown. In addition, xenograft tumor experiments showed that circIFT80 accelerated the tumorigenesis of CRC in vivo. In conclusion, our work reveals the impacts of circIFT80 as ceRNA in the progression of CRC, by which sponging miR-142, miR-568, and miR-634 enhanced the expression levels of *β*-catenin and activation Wnt/*β*-catenin pathway. Collectively, our data indicate that circIFT80 serves as an oncogene in CRC and represents a novel candidate for diagnosis and treatment.

## 1. Introduction

Colorectal cancer (CRC) is characterized by high rates of proliferation and metastasis and represents the third most common form of malignant tumor and the fourth leading cause of mortality in the world, thus creating a serious threat to health [1]. According to current global cancer statistics, there are almost 1.8 million new cases and 881,000 deaths due to CRC each year [2]. There are two main factors underlying the mortality associated with CRC: the high rates of recurrence and distant metastasis. Although current treatments for colorectal cancer have made great progress, including surgery, radiotherapy, and systemic chemotherapy; the survival rates of patients with CRC are still far from satisfactory [3, 4]. Therefore, there is an urgent need to identify the molecular mechanisms underlying the progression of CRC. Furthermore, the clear heterogeneity of CRC tumors indicates a clear need for new biological markers and therapeutic targets for CRC.

Noncoding RNAs, including circRNAs, microRNAs, and lncRNAs, have become a significant focus of research attention due to their crucial role in regulating the progression of multiple malignancies [5], including bladder cancer, gastric cancer, nasopharyngeal carcinoma, and pancreatic cancer. However, compared to the other forms of noncoding RNA, we know very little about the precise role of circRNA in cancer. circRNAs are covalently closed-loop structures that are usually very stable and not easily degraded in the cellular microenvironment [6]. circRNAs are usually located in the cytoplasm and can exert influence on the progression of various types of cancers; they can also serve as potential biomarkers and therapeutic targets for tumors, as demonstrated in multiple studies [7]. For example, circRNA_000166 can function as an oncogene by promoting the proliferation of CRC cells and by limiting apoptosis. circITGA7 serves as a tumor suppressor in CRC by inhibiting growth and metastasis by suppressing the Ras signaling pathway [8]. In most cases, circRNAs usually act as a competitive endogenous RNA (ceRNA) that can absorb miRNAs to regulate the expression of targeted genes [9]. Although research on circRNAs has advanced significantly, the precise relationship between circRNAs and CRC has yet to be elucidated. Further studies are now needed to elucidate the molecular mechanisms underlying circRNA action in the development and progression of cancers.

*β*-Catenin is a transcription factor that plays an important role in cell proliferation, cellular differentiation, apoptosis, and metastasis [10]. Previous research has demonstrated that *β*-catenin can contribute to malignant progression and distant metastasis in CRC by promoting proliferation, limiting apoptosis, and accelerating epithelial-mesenchymal transition [11, 12]. Previous researchers have demonstrated that miRNAs can also regulate the progression of CRC by targeting the mRNA of *β*-catenin. In the present research, we confirmed that *β*-catenin was simultaneously targeted by miR-142, miR-568, and miR-634 and that these were all targeted by circIFT80. In this study, we selected circiIFT80 (also referred to as circ0067835) by circRNA microarray sequencing technology for further investigation because of its abnormally high expression in CRC tissues and cells. circIFT80 can facilitate the growth and metastasis of CRC and inhibit apoptosis. We found that circIFT80 functioned as an oncogene to enhance the progression of CRC by regulating the repression of *β*-catenin by acting as a sponge for miR-142, miR-568, and miR-634. Our findings highlight a novel mechanism for CRC progression and identified a novel target for the diagnosis and treatment of CRC.

## 2. Materials and Methods

### 2.1. Cell Culture and Reagents

Human CRC cell lines (HT-29 and SW480), normal human colonic epithelial cells (HcoEpic), and 293 T cells were from the American Type Culture Collection (ATCC, Manassas, VA, USA). 293 T cells were SV-40 immortalized human renal uroepithelial cells which have been widely used as a tool cell in experiments. SW480, HcoEpic, and 293 T cells were maintained in DMEM culture medium (Gibco, Carlsbad, NY, USA) containing 10% fetal bovine serum (FBS, Gibco, Carlsbad, CA, USA) and 1% antibiotic-antimycotic (penicillin 100 U/mL and streptomycin 100 g/mL; HyClone Laboratories, Logan, UT). HT-29 cells were cultured in McCoy's 5A medium containing 10% FBS and 1% penicillin streptomycin solution. All cell types were cultured in an incubator at 37°C in 5% CO_2_ and 95% humidity in the confirmed absence of mycoplasma contamination. Cells were taken from early passages and used for experiments. CCK-8 solution was used to detect the viability of CRC cells and was purchased from TargetMol (Boston, USA). Annexin-V-FITC/PI Apoptosis Detection Kit was acquired from BD Biosciences (San Jose, CA, USA). *β*-Catenin and c-myc antibodies were purchased from Cell Signaling Technology (CST, Danvers, MA, USA), and a GAPDH antibody was purchased from Proteintech Group (Wuhan, China).

### 2.2. Tissue Collection

We collected CRC tissues from patients along with adjacent normal tissue (*n* = 18) from the Department of Gastrointestinal Surgery, Hainan General Hospital, Hainan Affiliated Hospital of Hainan Medical University. All clinical samples used in this study were approved by the Medical Ethics Committee of Hainan General Hospital (Med-Eth-Re [2020] 199), and informed consent was obtained in writing from the CRC patients (*n* = 18) who participated in this study. The study was conducted in accordance with the Declaration of Helsinki principles. The CRC tissues were obtained from the patients who had never experienced radiotherapy and systemic chemotherapy prior to surgery. The clinical information of the patients participating in the experiment is shown in Supplementary Table 1. All tissues were stored at -80°C prior to analysis.

### 2.3. circRNA Microarray Analysis

Total RNA was isolated from ten pairs of human CRC tissues and matched adjacent normal tissues using TRIzol Reagent (Invitrogen, Carlsbad, CA, USA) in accordance with the manufacturer's instructions. RNA was then quantified by the 260/280 ratio and a NanoDrop ND-1000 (Thermo Fisher Scientific, Wilmington, DE, USA). The example planning and microarray hybridization were performed according to Arraystar's standard protocols. The Arraystar Human circRNA Array (6x7K, Arraystar) was used to hybridize labeled cRNAs. The slides were washed, and the samples were analyzed by the Axon GenePix 4000B microarray scanner. The images were then imported into GenePix Pro 6.0 software (Axon) for grid alignment and data extraction. Quantile standardization and data handling were performed utilizing the R software bundle. Differentially expressed circRNAs with an adjusted *P* value ≤ 0.05 and a log FC ≥ 2 considered to be statistically significant. Differentially expressed circRNAs between the tumor and normal adjacent tissues were clustered in a Heatmap matrix. Meanwhile, annotations of the identified circRNAs were performed using the circBase and circ2Trait disease databases.

### 2.4. Cell Counting Kit-8 and Colony Formation Assays

Cell viability assays were conducted to evaluate the proliferation of CRC cells. The CCK-8 kits were used according to the manufacturer's protocols. In brief, CRC cells in the logarithmic growth phase were seeded into 96-well plates containing 5000 cells per well and incubated with the indicated conditions. After culturing for various durations (24 h, 48 h, 72 h, and 96 h), 10 *μ*l of CCK-8 solution was added into the 96-well plates with each well containing 100 *μ*l of complete medium. After incubation in an incubator for 2 h, then detect the absorbance at 450 nm with an ELISA reader (Bio-Rad, Hercules, CA, USA). For colony formation assays, the CRC cells were seeded in 6 cm dishes (1500 cells per dish) and then cultured in an incubator for at least 7 days to allow colony formation. Next, the cells in the 6 cm dishes were washed three times with PBS and then fixed and stained with 0.1% crystal violet dissolved with 4% paraformaldehyde for 10 min to make the cell colonies visible. The clonogenicity capacity of the CRC cells was then evaluated by enumerating the visible colonies on four random fields of vision under microscopy under 100x magnification. All researches were performed at least three times.

### 2.5. Cell Flow Cytometry

CRC cells were seeded into 6 cm dishes and incubated in a cell incubator for 48 h to increase the density of cells to approximately 80%. Then, the cells were harvested and suspended in PBS for subsequent experiments. For apoptotic analysis, the cells suspended in PBS were stained with 50 *μ*g/ml of propidium iodide (PI) and 50 *μ*g/ml of RNase A (1 : 1) staining solution for 15 min at room temperature. Finally, flow cytometry was performed to analyze the percentage of apoptotic CRC cells using a BD FACSCanto II (BD Biosciences, San Jose, CA, USA). For cell cycle analysis, the cells suspended in ice-cold PBS were fixed with 70% ethanol at 4°C at least 12 hours. After staining with 50 *μ*g/ml of propidium iodide (PI) and 50 *μ*g/ml of RNase A for 15 min in dark place, we used a FACSCalibur™ flow cytometer (BD Biosciences, USA) to evaluate the cell cycle distribution in each batch of CRC cells. This experiment was repeated in triplicate.

### 2.6. Wound Healing Assays

Wound healing assays were used to assess the migration ability of CRC cells with different expression levels of circIFT80. In brief, the cells were cultured in 6-well plates until the cell density reached approximately 100%. Then, two vertical scratches were created in the cells with a sterile 200 *μ*l pipette tip. CRC cells were washed with PBS to remove floating cells and then cultured with serum-free medium for 48 h. Finally, typical images were captured to allow us to observe changes of in the distance of single-layer cells under an inverted microscope (magnification, ×200) at 0 h and 48 h. We used the following formula to calculate the migration rate of CRC cells: (scratch width at 0 h − scratch width at 48 h)/scratch width at 0 h. These experiments were conducted in triplicate.

### 2.7. Quantitative RT-PCR

Total RNA was extracted with TRIzol™ Reagent (Invitrogen) and quantified with a microplate reader at 260 nm. For circRNA and mRNA, complementary DNA (cDNA) was synthesized by using 1000 ng RNA as a template and the Prime Script RT-PCR kit (Takara Bio Dalian, China). The expression of specific genes was then determined with a CFX96 Real-Time PCR system (Bio-Rad, CA, USA), SYBR-Green PCR Master Mix (Takara Bio, Dalian, China), and gene-specific primers. *18S* was utilized as internal control. The primers used in the research were shown as follows: *circIFT80*, F: CCGCCCACTGTACAATTCAC and R: TCTTCAGCAGTAGTCCAGCC; 18S, F: GTGGAGCGATTTGTCTGGTT and R: AACGCCACTTGTCCCTCTAA; and *β*-catenin, F: AAAGCGGCTGTTAGTCACTGG and R: CGAGTCATTGCATACTGTCCAT. For miRNAs, miRNA first-strand cDNA synthesis kit (Takara Bio Dalian, China) was used to generate cDNA for miRNA analysis and SYBR-Green PCR Master Mix (Takara Bio, Dalian, China) was also used to perform the quantitative real-time PCR assay with a CFX96 Real-Time PCR system (Bio-Rad, CA, USA). U6 was used as an internal control. The microRNA primer is shown in supplementary Table 2. Finally, the expression levels of these genes and miRNAs were calculated by the 2 ^−*ΔΔ*CT^ method and presented as a bar chart.

### 2.8. Western Blotting

CRC cells transfected with different constructs were harvested and incubated with RIPA Lysis and Extraction Buffer (Thermo Fisher Scientific) containing a protease inhibitor, phosphatase inhibitor, and 0.1 M PMSF. Total proteins were then extracted and quantified by BCA assay. Next, 20 *μ*g of each protein sample was separated by SDS-polyacrylamide gel electrophoresis (10%-15%) and transferred to PVDF membranes. After blocking with 10% nonfat milk for 1 h at room temperature and incubating with primary antibodies at 4°C, overnight. The specific antibodies are as follows: *β*-catenin (CST, USA), 1 : 1000 dilution; C-myc (Abcam, USA), 1 : 1000 dilution; MMP9 (Abcam, USA), 1 : 1000 dilution; MMP2 (Abcam, USA), 1 : 1000 dilution; and GAPDH (Abcam, USA), 1 : 5000 dilution; they were washed three times with1 × TBST(T: tris; B: buffer; S: solution; T: tween) for 15 min; the PVDF membranes were incubated in peroxidase-conjugated secondary antibody at room temperature for 1 h and then washed three times with 1 × TBST again. Finally, the ECL chemiluminescent detection system (Bio-Rad, USA) was used to determine protein expression levels.

### 2.9. Histology and Immunohistochemistry

Tumors were harvested from the nude mice, embedded in paraffin wax, and then cut into 5 *μ*m thick fragments for subsequent experiments. The paraffin sections were dewaxed in xylene, rehydrated in graded solutions of ethanol, heated for 5 min in a pressure cooker for antigen retrieval, and then incubated in 3% H_2_O_2_ for 10 min to block endogenous peroxidases. Nonspecific binding was blocked by the addition of 5% BSA (Bovine serum albumin). Next, the tissues were incubated at 4°C overnight with specific primary antibodies. The following antibodies were used: *β*-catenin (CST, USA), 1 : 200 dilution; C-myc (Abcam, USA), 1 : 100 dilution; and ki-67 (Abcam, USA), 1 : 500 dilution. The next day, the sections were washed and incubated with goat anti-rabbit IgG H&L/HRP antibody (1 : 3,000; BIOSS, China) at 37°C for 50 min. The images were photographed by an Olympus IX70 inverted microscope after staining with DAB.

### 2.10. Cell Transfection

siRNAs were designed to target the back-splice junction sequences of circIFT80 (si-circIFT80). We also designed a corresponding negative control siRNA (si-NC). The following are the target sequences of si-NC and circIFT80: circIFT80 siRNA 5′-GAGCCCAGGCAACUGAAGUAA-3′ and circIFT80 siRNA Control 5′-GAGCCCAGGCAACUGAUCAUU-3′; they are synthesized by GenePharma (Shanghai, China). The circIFT80 shRNA plasmid and scramble shRNA were designed and synthesized by GeneChem Company (Shanghai, China). pcDNA3.1, pcDNA-circIFT80, pcDNA-premiR-142, pcDNA-premiR-568, and pcDNA-premiR-634 plasmids were purchased from GeneCopoeia (Guangzhou, China) and the circIFT80 sequence information from circBank (http://www.circbank.cn/index.html, circBase ID: hsa_circ_0067835). The miRNA mimic used for the overexpression of miRNA was purchased from Ribobio (Guangzhou, China). Lipofectamine 3000 reagent (Invitrogen, Carlsbad, USA) was used for transfection in accordance with the manufacturer's instructions.

### 2.11. Dual-Luciferase Reporter Assay

We used the CircInteractome database (https://circinteractome.nia.nih.gov) for bioinformatics analysis. First, we identified the binding sites between miR-142, miR-568, miR-634, and circIFT80 as well as miR-142, miR-568, miR-634, and *β*-catenin. The circular RNA IFT80 and *β*-catenin sequences, containing the miR-142, miR-568, and miR-634 binding sites, were inserted into luciferase reporter vectors (WT-circIFT80, WT-*β*-catenin). Then, site-specific mutated versions of the miR-142, miR-568, and miR-634 binding sites in the circIFT80 and *β*-catenin sequences were also inserted into luciferase reporter vectors (MUT-circIFT80, MUT-*β*-catenin). 293 T cells were cotransfected with miR-142, miR-568, miR-634 mimic, or NC mimic, with WT-circIFT80/MUT-circIFT80 or WT-*β*-catenin/MUT-*β*-catenin using Lipofectamine 3000. Transfected cells were cultured for 48 h, and then luciferase activity was detected with a Dual-Luciferase Reporter Assay System (Promega, Madison, WI, USA). Renilla luciferase was used as an internal control.

### 2.12. RNA Pull-Down Assays

Pierce™ Magnetic RNA-Protein Pull-Down Kit (Thermo Scientific) were used RNA pull-down assay. Biotin-labeled miR-142, miR-568, and miR-634 probes, or a corresponding miRNA control probe, were synthesized by GenePharma (Shanghai, China). The probe sequence is shown in supplementary Table 3. HT-29 cells were lysed with lysis solution, treated with biotin-labeled miR-142, miR-568, and miR-634 probes, and incubated with streptavidin agarose beads at 4°C overnight. Next, the beads were collected and washed three times. The RNA complexes were then isolated with TRIzol™ Reagent (Invitrogen) and analyzed by quantitative RT-PCR assay.

### 2.13. circIFT80 and miRNA Colocation Assay

The circIFT80 subcellular localization assay was performed as described previously [13]. In brief, HT29 cells were cotransfection with pcDNA-circIFT80 and pcDNA-premiR-142 (orpcDNA-premiR-568, orpcDNA-premiR-634) expressing vectors, and the cells were paraformaldehyde fixation at the time of exponential phase and were 80–95% confluent. After prehybridization, biotin-labelled probes specific to circIFT80 (GenePharma (Shanghai), China) and Dig-labelled miR-142 probes (GenePharma (Shanghai), China) were used in the hybridization. The signals of biotin-labelled probes were detected using Cy5-Streptavidin (Life Technologies). The signals of Dig-labelled locked nucleic acid miR-124 probes were detected using tyramide-conjugated Alexa 488 fluorochrome TSA kit (Life Technologies). Nuclei were counterstained with 4,6-diamidino-2-phenylindole (DAPI). The images were obtained with a confocal microscope (Olympus, USA).

### 2.14. Xenograft Tumor Model

All male athymic nude mice used in this study were approved by the Ethical Committee of the Hainan General Hospital, Hainan Affiliated Hospital of Hainan Medical University (Approval number: SYXK 2020-0012). Twelve male athymic nude mice (BALB/c-nu/nu), 4-weeks of age, weighing approximately 16-18 g were randomly divided into two groups with six mice per group. The mice were raised in sterile conditions with adequate water and feed for one week. Subsequently, the mice were subcutaneously injected in both flanks with approximately 1 × 10^6^ HT-29 cells (sh-NC or sh-circIFT80). A vernier caliper was then used to measure the length (*L*, mm) and weight (*W*, mm) of tumor xenografts on a weekly basis. Tumor volume was calculated according to the following formula: *L* (mm) × *W*^2^ (mm^2^)/2. After five weeks, the mice were euthanized by cervical dislocation to harvest tumors. The tumors were weighed and fixed in 4% paraformaldehyde for subsequent experiments. Immunohistochemistry assays were performed to detect the expression levels of specific proteins.

### 2.15. Statistical Analysis

All statistical analyses were carried out with GraphPad Prism 8.0 software (GraphPad Software, Inc., La J olla, CA, USA) and two-tailed Student's (*t*-test). Data are presented as the mean ± standard deviation (SD) of three independent experiments. *P* < 0.05 was regarded as the threshold value for statistical significance.

## 3. Results

### 3.1. circIFT80 Was in Abnormally High Expression in CRC Tissues and Cells

**Figure 1:**
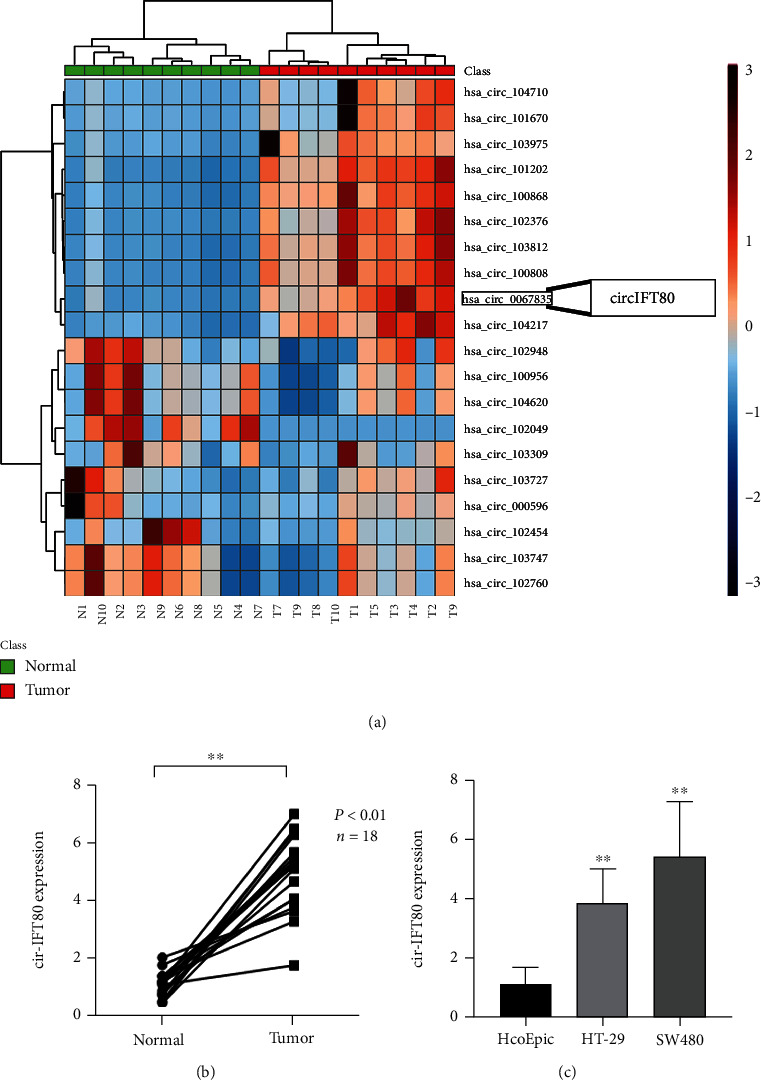
circIFT80 was in abnormally high expression in CRC tissues and cells. (a) circIFT80 was highly expressed in CRC tissues compared with adjacent normal tissue, as determined by microarray analysis. (b) Quantitative PCR (qPCR) analysis of the relative expression levels of circIFT80 in normal tissues and CRC tissues; paired *t*-test; ^∗∗^*P* < 0.01. (c) Quantitative PCR (qPCR) analysis of the circIFT80 relative expression level in both CRC cells (HT-29 and SW480) and normal colonic cell (HcoEpic). ^∗∗^*P* < 0.01.

circRNA microarray sequencing technology revealed that circIFT80 (also referred to as has_circ_0067835) was upregulated in10 CRC tissues when compared with corresponding adjacent normal tissues (Figure 1(a)). We had conducted some preliminary experiments on the function of circIFT80 in CRC and found that circiIFT80 sever as an oncogene in CRC. When combined with the results of sequencing, we selected circIFT80 as the candidate for further studies in this research. Then, we investigated the expression levels of circIFT80 in CRC tissues and cells. The detection of circIFT80 expression in 18 pairs of CRC tissues and matched normal tissues demonstrated that circIFT80 was highly expressed in colorectal cancer tissues (Figure 1(b)). We also detected the expression levels of circIFT80 in CRC cells (HT-29 and SW480) and normal human colonic epithelial cells (HcoEpic). We found that circIFT80 was upregulated in CRC cells (Figure 1(c)). These results indicated that circiIFT80 was commonly upregulated in the CRC tissues and cells studied herein.

### 3.2. circIFT80 Overexpression Facilitated the Proliferation and Colony Formation of CRC Cells

**Figure 2:**
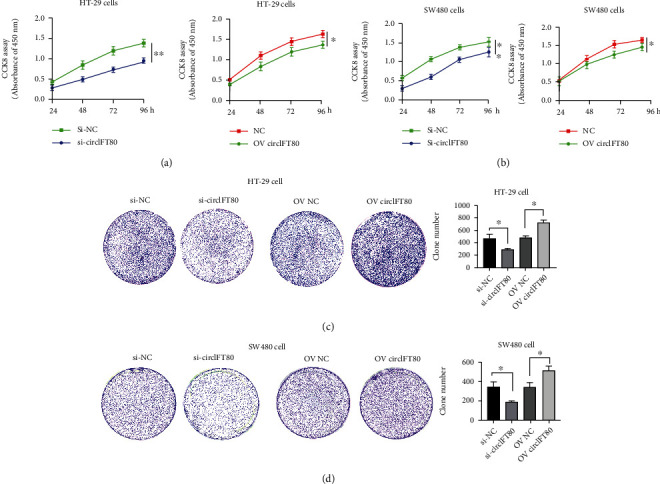
circIFT80 overexpression facilitated the proliferation and colony formation of CRC cells. (a) Cell viability of CRC cells as detected by CCK-8 assays following transfection with si-circIFT80 or OE circIFT80 into HT-29 cells. (b) Cell viability of CRC cells was detected by CCK-8 assays transfection with si-circIFT80 or OE circIFT80 into SW480 cells. (c) Colony formation assays were conducted in CRC cells transfected with si-circIFT80 or Ov-circIFT80 into HT-29 cells. (d) Colony formation assays were conducted in CRC cells transfected with si-circIFT80 or Ov-circIFT80 into SW480 cells. ^∗^*P* < 0.05 and^∗∗^*P* < 0.01. si-circIFT80: circIFT80 silencing; si-NC: the control sequence of si-circIFT80; Ov-circIFT80: overexpressed circIFT80; NC: pcDNA3.1 plasmid.

Our previous results confirmed that circIFT80 was highly expressed in colorectal cancer. Next, we investigated the biological function of circIFT80 in CRC based on gain-of-function and loss-of-function experiments. We used small interfering RNAs (siRNAs) targeting the back-splice sequence of circIFT80 to inhibit the expression of circIFT80, and a pcDNA-circIFT80 expression vector was used to establish the ectopic overexpression of circIFT80. The CCK-8 assay was used to determine the viability of CRC cells. The data showed that circIFT80 silencing (si-circIFT80) significantly inhibited the proliferation both of HT-29 and SW480cells, whereas the overexpression of circIFT80 (Ov-circIFT80) promoted the proliferation of CRC cells (Figures 2(a) and 2(b)). Colony formation assays demonstrated the inhibition of si-circIFT80on HT-29 cells and SW480 cells and the promotion of Ov-circIFT80 on CRC cell proliferation. (Figures 2(c) and 2(d)). These data indicated that circIFT80 promoted cell proliferation and colony formation.

### 3.3. circIFT80 Facilitated the Ability of CRC Cells to Migrate

**Figure 3:**
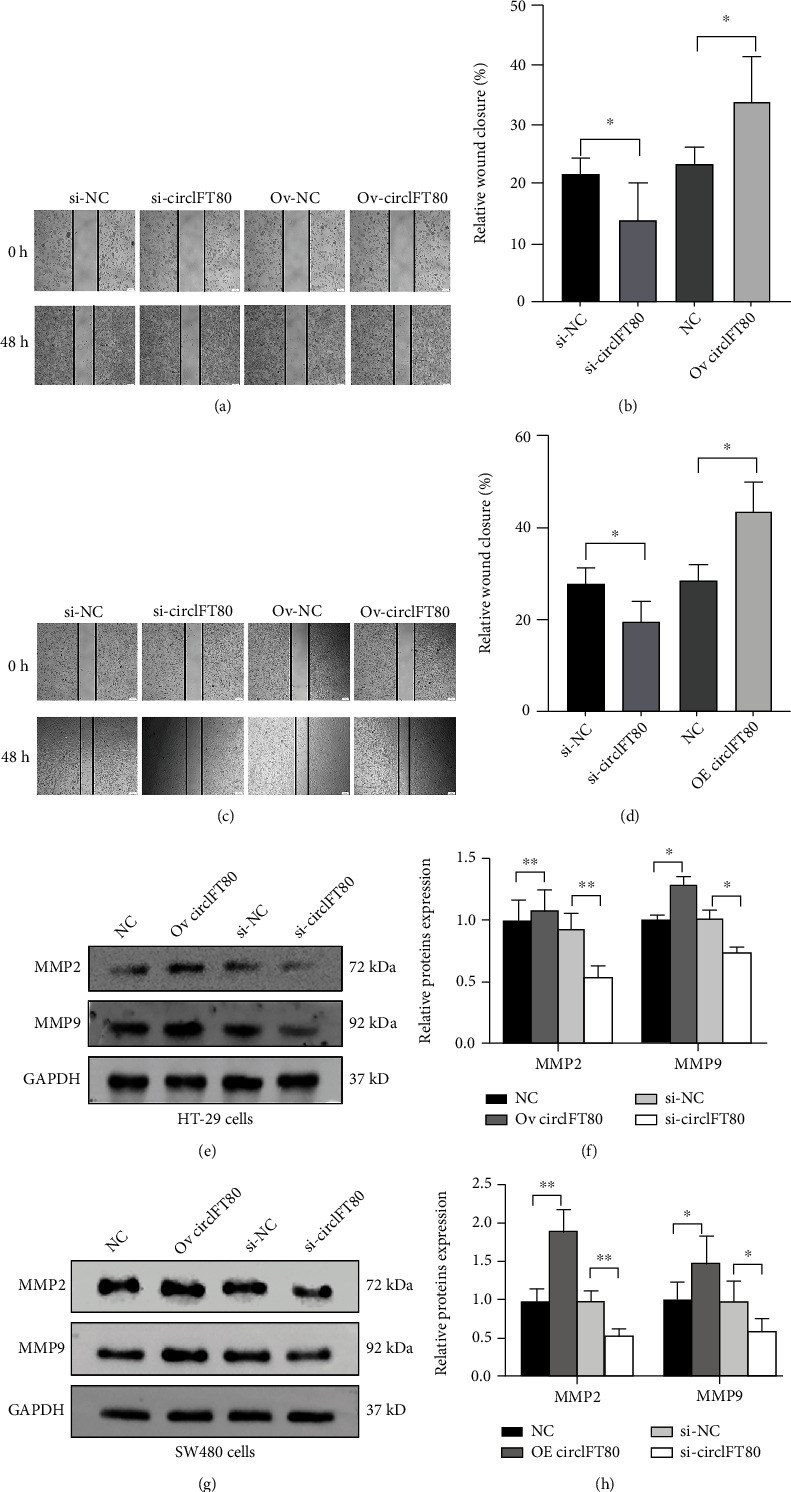
circIFT80 facilitated the ability of CRC cells to migrate. (a, b) Wound healing assays in HT-29 cells transfected with si-circIFT80 or Ov-circIFT80. Quantitative data is shown below. (c, d) Wound healing assays in SW480 cells transfected with si-circIFT80 or Ov-circIFT80. Quantitative data is shown below. Quantitative data is shown below. (e, f) The protein level of MMP2 and MMP9 was measured by western blot in HT-29 cells with si-circIFT80 or Ov-circIFT80. (g, h) MMP2 and MMP9 expression was detected by a western blot in SW480 cells with si-circIFT80 or Ov-circIFT80. ^∗^*P* < 0.05 and^∗∗^*P* < 0.01. The data from three independent experiments.

Next, we used the wound healing assay to evaluate the potential role of circIFT80 on the ability of CRC cells to migrate *in vitro*. We found that migration ability was significantly suppressed in si-circIFT80 cells compared with si-NC cells, while on the migration capacity of CRC cells overexpressing circIFT80 was significantly higher than that of negative control cells (Figures 3(a) and 3(b)). MMP2 and MMP9, as the two most important members of the matrix metalloproteinases, play a crucial role in promoting cancer metastasis [14]. We found that the MMP2 and MMP9 protein levels were suppressed in HT-29 cells and SW480 cells after circIFT80 was knocked down. And the MMP2 and MMP9 protein levels were upregulated after overexpression of circIFT80 (Figure 3(c)). These results clearly indicated that circIFT80 facilitated the migration ability of CRC cells *in vitro*.

### 3.4. circIFT80 Contributed to Cell Cycle Progression and Reduced Cellular Apoptosis

**Figure 4:**
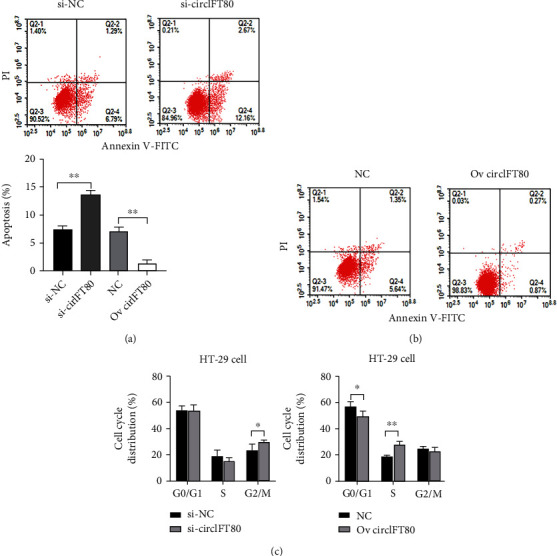
circIFT80 contributed to cell cycle progression and reduced cellular apoptosis. (a) The extent of apoptosis in CRC cells was detected by flow cytometry following the transfection of si-NC and si-circIFT80. (b) The extent of apoptosis in CRC cells was detected by flow cytometry following transfection with Ov-circIFT80 or NC. Quantitative results are shown in the left panel. (c) The cell cycle distribution of CRC cells was analyzed by flow cytometry. Quantitative data are shown below. ^∗^*P* < 0.05 and^∗∗^*P* < 0.01.

The malignant proliferation of tumor cells is closely related to poor prognosis and is characterized by accelerated cell cycle progression and reduced levels of apoptosis. Hence, the carcinogenic effect of circIFT80 on cell cycle progression and cellular apoptosis was detected by flow cytometry. We found that the loss of circIFT80 increased the proportion of apoptotic CRC cells, while the ectopic overexpression of circIFT80 reduced the proportion of apoptotic CRC cells (Figures 4(a) and 4(b)). The loss of circIFT80 also caused cell cycle arrest in the G2/M phase in CRC cells. Correspondingly, the ectopic overexpression of circIFT80 contributed to the acceleration of G1-phase to S phase transition in CRC cells (Figure 4(c)). These results suggested that circIFT80 contributed to cell cycle progression and cell apoptosis reduction in CRC cells.

### 3.5. *β*-Catenin Expression Was Regulated by circIFT80 in CRC Cells

**Figure 5:**
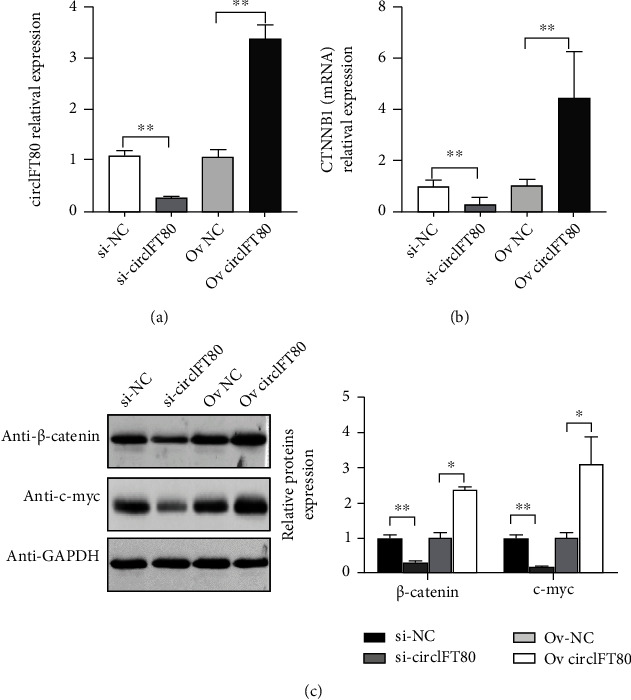
*β*-Catenin expression was regulated by circIFT80 in CRC cells. (a) The expression levels of circIFT80 were analyzed by qPCR in HT-29 cells transfected with si-NC, si-circIFT80, pcDNA3.1, and pcDNA-circIFT80. (b) The expression levels of *β*-catenin (CTNNB1) were measured by qPCR in HT-29 cells transfected with si-NC, si-circIFT80, pcDNA3.1, and pcDNA-circIFT80. (c) The protein levels of *β*-catenin and c-myc were measured by western blotting in HT-29 cells transfected with si-NC, si-circIFT80, pcDNA3.1, and pcDNA-circIFT80. Quantitative data are shown in the right panel. GAPDG was used as a loading control. ^∗∗^*P* < 0.01 and^∗∗∗^*P* < 0.001.

The *β*-catenin pathway plays a crucial role in regulating the proliferation, migration, and metastasis of cancers [15]. Hence, we investigated the specific relationship between circIFT80 and *β*-catenin. Firstly, cells with different circiFT80 expression levels were constructed with si-circIFT80 or pcDNA-circIFT80 for subsequent gain-of-function and loss-of-function experiments (Figure 5(a)). The mRNA and protein levels of *β*-catenin, and its downstream target gene c-myc, were investigated by qPCR and western blotting, respectively. We found that the inhibition of circITF80 expression levels was followed by a reduction in *β*-catenin expression, while the overexpression of circIFT80 was followed by an increase in *β*-catenin expression. The expression levels of c-myc, the downstream target gene of *β*-catenin, changed in accordance with *β*-catenin (Figures 5(b) and 5(c)). These results suggest that *β*-catenin was regulated by circIFT80 and may serve as the target gene of circIFT80 in CRC cells.

### 3.6. circIFT80 Acted as a Molecular Sponge for miR-142, miR-568, and miR-634 in CRC Cells

**Figure 6:**
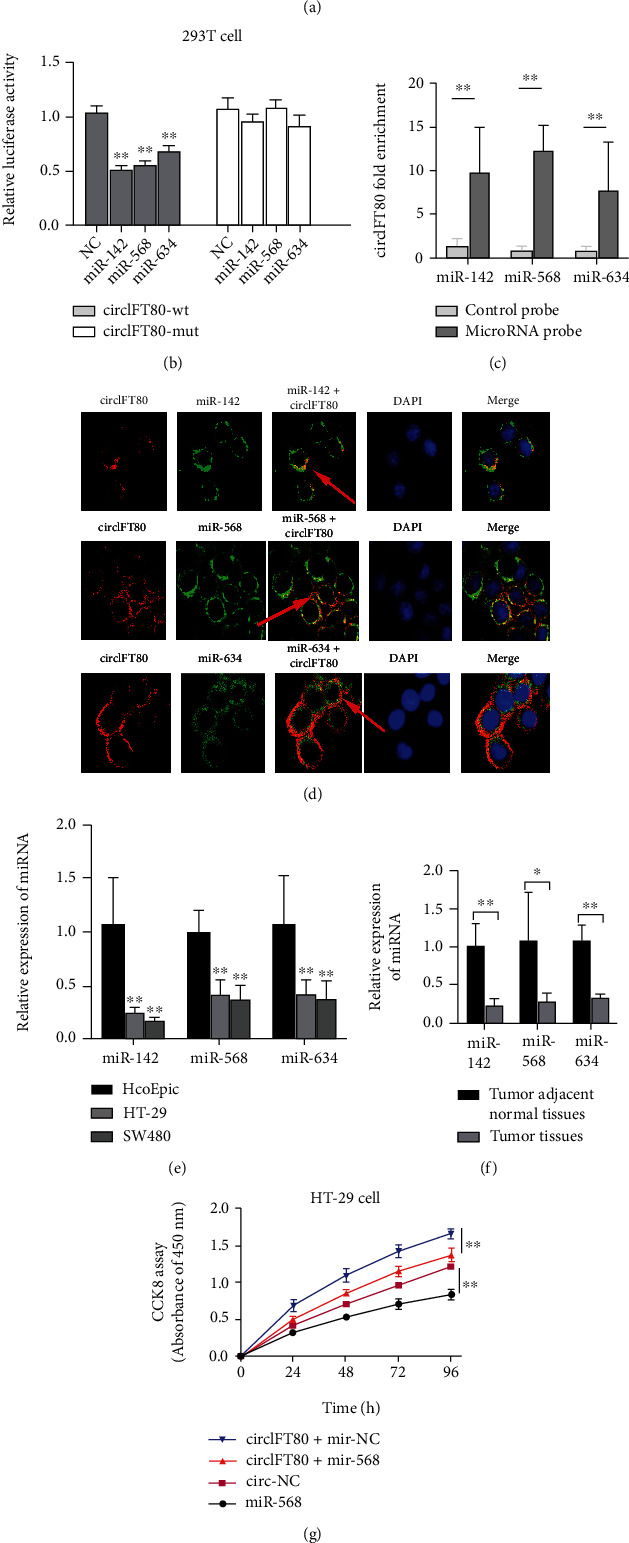
circIFT80 acted as a molecular sponge for miR-142, miR-568, and miR-634 in CRC cells. (a) circIFT80 containing wild-type (WT) or mutant (MUT) binding sites along with the sequence complementarity between miR-142, miR-634, and miR-568. (b) Relative luciferase activity was detected in 293 T cells after cotransfection with the WT or MUT circIFT80 reporter plasmids in NC or miR-142, miR-634, and miR-568 mimic. (c) circIFT80 expression levels were analyzed by qPCR after RNA pull-down assays using biotin-labeled miR-142, miR-568, and miR-634 probes and a control probe. (d) In situ hybridization assay of the colocation of circIFT80 and miR-142, miR-568, and miR-634. (e) The expression level of miR-142, miR-568, and miR-634 between CRC cells (HT-29 and SW480) and normal human colonic epithelial cells (HcoEpic) was analyzed by qPCR assay. (f) The expression level of miR-142, miR-568, and miR-634 between CRC tissues and tumor adjacent normal tissues was detected by qPCR assay. (g) The cell viability of HT-29 cells following different transfections, as evaluated by CCK-8 assays. U6 was used as an internal control. ^∗^*P* < 0.05 and^∗∗^*P* < 0.01.

Previous studies have demonstrated that circIFT80 is predominantly located in the cytoplasm, suggesting that it exerts its procancer effects mainly through the action of ceRNA. We demonstrated that *β*-catenin may be a target gene for the carcinogenic effect of circIFT80. Hence, we used the Starbase v3.0 target prediction tool (http://starbase.sysu.edu.cn/) to predict the miRNAs that could be targeted by circIFT80 and could also simultaneously target *β*-catenin mRNA (CTNNB1). After identifying the intersection, we selected miR-142, miR-568, and miR-634 for subsequent experimental verification. Next, we used the Circular RNA Interactome (https://circinteractome.nia.nih.gov/index.html) to forecast the potential interaction between circRNA and miRNA. The binding sites between circIFT80 and miR-142, miR-568, and miR-634 are shown in Figure 6(a). To further verify the targeting relationship between circRNA and its targeted miRNA, we performed a dual luciferase reporter assay. The results showed that luciferase intensity was suppressed following cotransfection with the wild-type circIFT80 and miR-142, miR-568, and miR-634 mimics, while the mutated version of circIFT80 exhibited no such effect (Figure 6(b)). In a further RNA pull-down experiment, transfection with biotin-labeled miR-142, miR-568, and miR-634 probes was accompanied by the enrichment of circIFT80, the biotin-labeled control showed no such effect (Figure 6(c)). Next, we found that the circIFT80 and miR-142, miR-568, and miR-634 were colocated in the cytoplasm by in situ hybridization assay (Figure 6(d)). These results confirmed the target binding relationship between circIFT80 and miR-142, miR-568, and miR-634. We have confirmed that circIFT80 is highly expressed in CRC, and then, we investigated the expression level of miR-142, miR-568, and miR-634 in CRC tissues and cells. The result showed that the expression of miR-142, miR-568, and miR-634 was downregulated in CRC tissues compared with tumor adjacent normal tissues and downregulated in CRC cells compared with the normal human colonic epithelial cells (HcoEpic cell) (Figures 6(e) and 6(f)). CCK-8 assays also demonstrated that miR-568 served as an antioncogene in CRC cells and that the tumor-promoting action of circIFT80 overexpression could be partly reversed by the overexpression of miR-568 (Figure 6(g)). These results demonstrated that circIFT80 exerted its proproliferative, migration, and carcinogenic effects by targeting and sponging miR-142, miR-568, and miR-634.

### 3.7. *β*-Catenin Was a Direct Target of miR-142, miR-568, and miR-634

**Figure 7:**
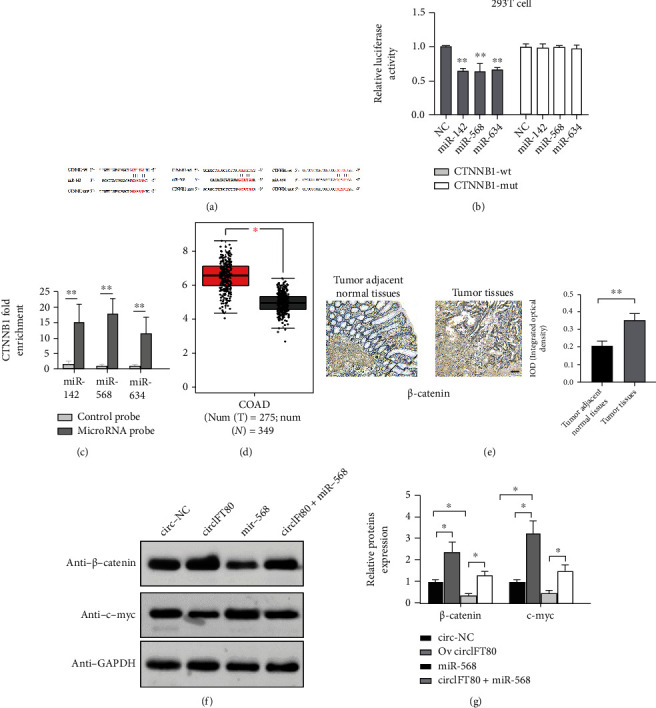
*β*-Catenin was a direct target of miR-142, miR-568, and miR-634. (a) The 3′UTR regions of CTNNB1 (*β*-catenin mRNA) containing wild-type (WT) or mutant (MUT) binding sites along with the sequence complementarity between miR-142, miR-634, and miR-568. (b) Relative luciferase activity was detected in 293 T cells after cotransfection with WT or MUT CTNNB1 reporter plasmids in NC or miR-142, miR-634, and miR-568 mimic. (c) CTNNB1 expression levels were analyzed by qPCR after RNA pull-down assays with biotin-labeled miR-142, miR-568, and miR-634 probes and a control probe. (d) The mRNA relative expression level of *β*-catenin in CRC and adjacent normal tissues in GEPIA downloaded from the TCGA. (e) *β*-Catenin expression in six pairs of CRC tissues and corresponding tumor adjacent normal tissues as detected by IHC assays. Quantification analysis is shown in the right panel. (f, g) The protein levels of *β*-catenin and c-myc were analyzed by western blotting in HT-29 cells following different transfections. The scale bar represents 100 *μ*m. ^∗^*P* < 0.05 and^∗∗^*P* < 0.01.

Using Starbase V3.0, we found that *β*-catenin (CTNNB1) might be the target gene for miR-142, miR-568, and miR-634. The binding sites between *β*-catenin and miR-142, miR-568, and miR-634 are shown in Figure 7(a), as determined by TargetScanHuman version 7.2 (http://www.targetscan.org/). To further verify the targeting relationship between *β*-catenin and these miRNAs, we performed a dual luciferase reporter assay. We found that luciferase intensity was suppressed following cotransfection of the wild-type *β*-catenin (CTNNB1) and miR-142, miR-568, and miR-634 mimics, while the mutated version of *β*-catenin (CTNNB1) exhibited no such effect (Figure 7(b)). In a further RNA pull-down experiment, transfection with biotin-labeled miR-142, miR-568, and miR-634 probes was accompanied by the enrichment of *β*-catenin (CTNNB1); the biotin-labeled control exhibited no such phenomenon (Figure 7(c)). Meanwhile, we analyzed the expression of *β*-catenin in the COAD (Colon adenocarcinoma) dataset using GEPIA (http://gepia.cancer-pku.cn/) for The Cancer Genome Atlas (TCGA). We found that *β*-catenin expression was significantly higher in 275 COAD (colon adenocarcinoma) tissues than in 349 normal control tissues (Figure 7(d)). Consistently, IHC assays also demonstrated that *β*-catenin was highly expressed in CRC tissues when compared with adjacent normal tissues (*n* = 6) (Figure 7(e)). Finally, we also verified that miR-568 suppressed the expression of *β*-catenin and c-myc and that the upregulation of *β*-catenin and c-myc induced by the overexpression of circIFT80 could be partly reversed by the overexpression of miR-568 (Figures 7(f) and 7(g)). These data suggested the existence of a regulatory network involving the circIFT80/miR-142, miR-634, miR-568/*β*-catenin pathways.

### 3.8. circIFT80 Depletion Suppressed the Tumorigenicity of CRC *In Vivo*

**Figure 8:**
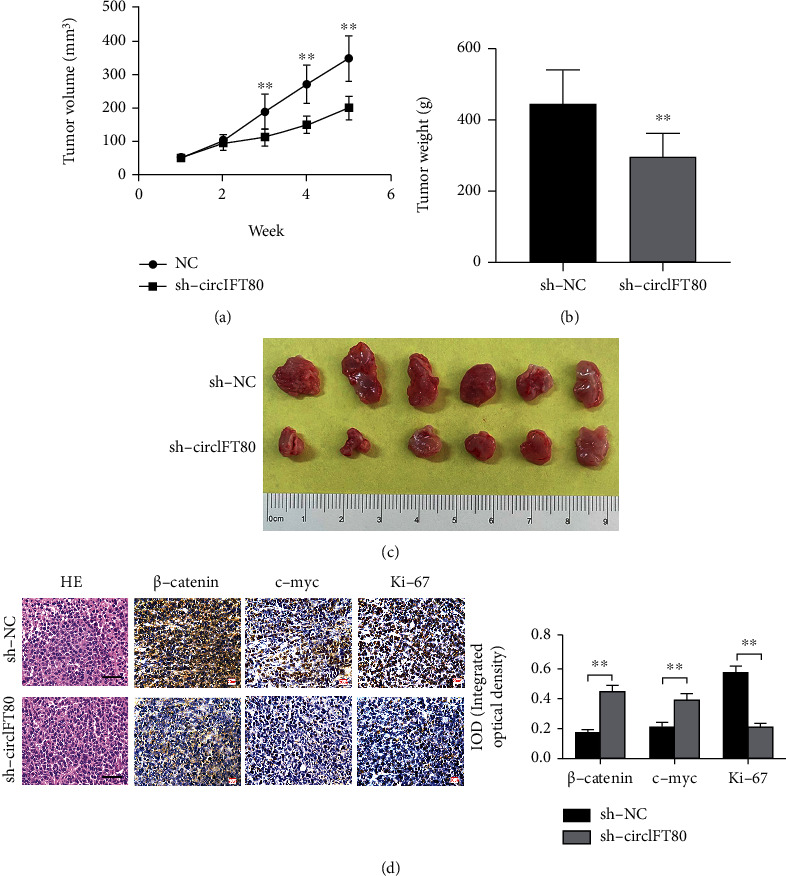
circIFT80 depletion suppressed the tumorigenicity of CRC in vivo. (a) Changes in tumor volume in the sh-circIFT80 and NC groups were measured weekly during the experiment. (b) The wet weights of tumors in the nude mice were measured after the nude mice had been sacrificed. (c) Representative images of the tumors in the NC and sh-circIFT80 groups are shown in the right panel. (d) Immunohistochemistry was used to analyze the expression of *β*-catenin, c-myc, and Ki-67 (*N* = 6). Quantification analysis is shown in the right panel. The scale bar represents 50 *μ*m. ^∗∗^*P* < 0.01.

Finally, the tumorigenicity of circIFT80 was validated in a xenograft model. In the preliminary experiment *in vivo*, we found that the tumorigenicity of HT-29 cell *in vivo* was significantly higher than that of SW480. Therefore, although circIFT80 was expressed at a higher level in SW480, we still chose HT-29 cell to establish the xenograft tumor model. Here, we injected circIFT80-depleted or negative control HT-29 cells in both flanks of nude mice. And the following volumes of the tumor in each mouse were measured weekly for 5 weeks; after sacrifice, weighed the harvested tumors. The changes in tumor volumes in the circIFT80-depleted and negative control groups are shown in Figure 8(a) while changes in tumor weight are shown in Figures 8(a) and 8(b). These results demonstrated that the circIFT80 knockdown group xenograft tumors grew more slowly than the control group. Consistently, the tumor weight in the circIFT80-depleted group was smaller. Representative images of tumors are shown in Figure 8(c). Meanwhile, there was no significant difference in body weight change between the two groups (Supplementary Figure 1). The expression of Ki-67, *β*-catenin, and c-myc was also investigated by IHC assay. It was shown that circIFT80 knockdown could suppress the expression of *β*-catenin, c-myc, and Ki-67 in xenograft tumor tissues (Figure 8(d)). These data suggested that circIFT80 serves as an oncogene in CRC cells and that circIFT80 knockdown attenuated the tumorigenicity of CRC *in vivo*.

## 4. Discussion

CRC is one of the most common malignant tumors of the digestive system and has the third highest mortality and morbidity rate worldwide [16]. Considering that most patients with CRC have no apparent symptoms in the early stages, 60% of them progress to intermediate or advanced stages with lymph nodules and distant metastasis [17]. Despite the widespread use of radical tumor resection and adjuvant radiotherapy in clinical practice, CRC still has a recurrence rate of 25-40% [18]. It is particularly important to understand the pathogenesis and molecular biology underlying the development of CRC. Therefore, the identification of new biomarkers for CRC is crucial in terms of recurrence, diagnosis, and treatment.

circRNA is a novel class of noncoding RNA that has become a significant research focus over recent years and features a closed continuous loop that is formed by covalent attachment of the 3′ and 5′ ends, thus making this form of RNA more stable and more resistant to exonucleases when compared with linear RNA isoforms [19]. As early as the 1970s, Sanger et al. [20] discovered the existence of single-stranded circRNA. However, the existence of circRNAs did not receive sufficient attention and recognition for many years due to limitations relating to detection and recognition. Over recent years, an increasing number of studies have found that circRNAs play a crucial biological regulatory role in various diseases, especially in malignancies, such as bladder, thyroid, prostate, and breast cancer. circRNAs are also known as a regulator for cell biological behavior, including cell proliferation, cell cycle progression, distant metastasis, and cell apoptosis [20]. Nowadays, circRNAs are increasingly being considered as biological markers and therapeutic targets for cancer due to their stable structure and specific bioregulatory functions. In this study, we demonstrated that circIFT80 functions as an oncogene and can be considered a target for the diagnosis and therapy of CRC.

Two previous studies have examined the role of circIFT80 in CRC. For example, circIFT80 was found to function as a ceRNA for miR-1236-3p to promote the progression of CRC [21]. Exosomal circIFT80 was also shown to enhance tumorigenesis and suppress radiosensitivity in CRC by regulating the miR-296-5p/MSI1 axis [22]. In our research, we found that circIFT80 was aberrantly overexpressed in CRC tissues when compared with normal adjacent tissues, as determined by circRNA microarray sequencing technology. We also demonstrated the elevated expression of circIFT80 in CRC patient tissues and CRC cells. Furthermore, the overexpression of circIFT80 was shown to promote cell proliferation, cell cycle progression, and migration but inhibited apoptosis in CRC cells; knockdown resulted in directly opposite effects. The upregulation of circIFT80 also increased the tumorigenicity of CRC *in vivo*. These findings suggested that circIFT80 functions as an oncogene in CRC progression.

circRNAs exert their bioregulatory functions in cancer *via* a range of different mechanisms, including acting as miRNA sponges, interacting with proteins, translating proteins or peptides, and regulating gene splicing or transcription, and by epigenetic regulation [23]. circRNAs are mainly located in the cytoplasm and predominantly act as miRNA sponges, thus influencing the expression of microRNAs and target genes; in turn, these effects exert a vital role in disease. For instance, hsa_circRNA_101996 promotes the proliferation and invasion of cervical cancers by sponging miR-8075 and by activating TPX2 expression [24]. circRNA_100269 acts as an antioncogene in gastric cancer and inhibits tumor cell growth by sponging miR-630 [25]. Furthermore, circ0082182 promotes cell proliferation, cell cycle progression, migration, invasion, and EMT (epithelial-mesenchymal transition) and inhibits cell apoptosis in CRC by simultaneously sponging miR-411 and miR-1205. The present, RNA pull-down, dual-luciferase reporter assays, and circIFT80 withmiR-142, miR-568, and miR-634co-location in cytoplasm demonstrated the sponge effects of circIFT80 on miR-142, miR-568, and miR-634. Contrary to the high expression of circIFT80 in CRC, the expression of miR-142, miR-568, and miR-634 in CRC tissues and cells was downregulated which also suggested there was a negative functional regulatory relationship between the circIFT80 and miRNAs (miR-142, miR-568, and miR-634). miR-142 has been also demonstrated to act as a tumor suppressor in CRC to suppress proliferation, metastasis, and tumorigenicity [26, 27]. Therefore, we can draw a conclusion that the carcinogenic action of circIFT80 is achieved by serving as a sponge for miR-142, miR-568, and miR-634.

It is well known that proteins are fundamental to biological functionality and that circRNAs and miRNAs both regulate tumor progression by binding to mRNAs and thus regulating protein expression levels [28]. The role of the *β*-catenin signaling pathway in key cellular processes in tumors, such as cell proliferation, migration, and differentiation, is well documented [29]. *β*-Catenin can also be regulated by both miRNA and circRNA to exert procancer effects. For instance, has-circ008494 promotes the carcinogenesis and progression of papillary thyroid carcinoma by targeting the miR-876-3p/ctnnb1 axis [30]. has-circ0002577 facilitates the progression of endometrial carcinoma by regulating the miR-197/ctnnb1 axis [31]. Dual-luciferase reporter and RNA pull-down assays carried out in the present study demonstrated that *β*-catenin was simultaneously regulated and targeted by miR-142, miR-568, and miR-634; the relationship between *β*-catenin and miR-142 has also been demonstrated in previous research [32]. Bioinformatics data further showed that *β*-catenin was highly expressed in CRC tissues and that *β*-catenin expression was negatively correlated with miR-568 expression and positively correlated with circIFT80 expression in CRC. The overexpression of miR-568 partly abolished the carcinogenic effects of circIFT80 in CRC cells. Collectively, our data suggested that circIFT80 regulates the progression of CRC by targeting miR-568, miR-142, and miR-634 to modulate *β*-catenin expression.

## 5. Conclusions

We demonstrated that circIFT80 was overexpressed in CRC and can sponge miR-142, miR-568, and miR-634, to promote the progression of CRC by regulating the levels of *β*-catenin. Collectively, our data indicate that circIFT80 serves as an oncogene in CRC and represents a novel candidate for diagnosis and treatment.

The current study is not without its limitations. The expression of circIFT80 was significantly higher in 18 pairs of colorectal cancer tissues when compared to adjacent normal tissues. However, the conclusion that circIFT80 can be used as a diagnostic marker for colorectal cancer from 18 pairs of clinical samples is not sufficient. Therefore, increasing the sample size for testing is also a priority for future work. Although we have made some important findings relating to the effect and underlying molecular mechanisms of circIFT80 in CRC, the precise effect of circIFT80 in regulating the progression of CRC still needs to be further explored. For instance, we need to ascertain the identity of the specific signaling pathways downstream of circIFT80/miR-142, miR-568, and miR-634/*β*-catenin and identify how these pathways are regulated in CRC. It is also important to ascertain whether the aberrant expression or regulation of circIFT80 can be detected and corrected in clinical practice. These limitations will be the focus of future research.

## Data Availability

The datasets used or analyzed during the current study are available from the corresponding authors on reasonable request.

## Abbreviations

CRC: Colorectal cancer
circRNAs: Circular RNAs
PMSF: Phenylmethanesulfonyl fluoride
PVDF: Polyvinylidene fluoride.

## Supplementary Materials

Supplementary 1 Supplementary Figure 1: after subcutaneously injecting in both flanks the HT-29 cells (sh-NC or sh-circIFT80), the mouse body weight changes in the sh-NC, and sh-circIFT80 groups were measured and recorded per week.

Supplementary 2 Table 1: the clinical information of the patients participating in the experiment. Table 2: primer sequences used in quantitative RT-PCR. Table 3: biotin-labeled miRNA probes.
