# Supplementary figures and images for: circIFT80 Functions as a ceRNA for miR-142, miR-568, and miR-634 and Promotes the Progression of Colorectal Cancer by Targeting β-Catenin
Source: Dis Markers. 2022 Jun 23;2022:8081246. doi: 10.1155/2022/8081246 (PMC9247842; doi:10.1155/2022/8081246)

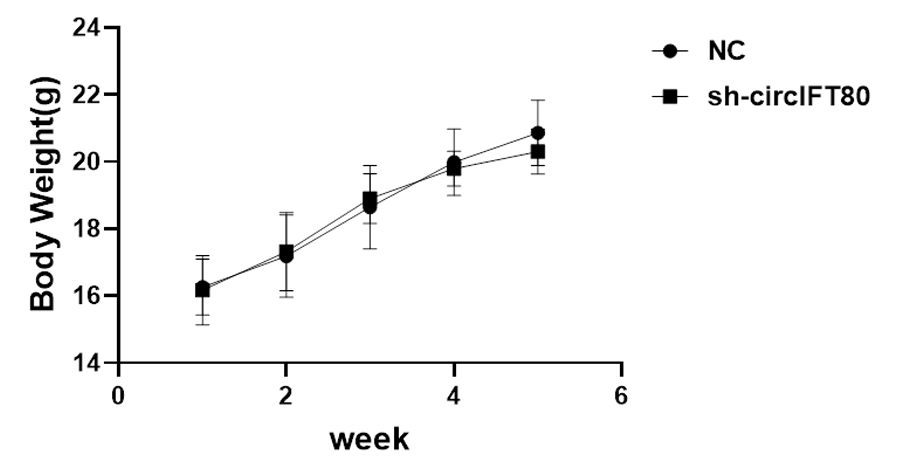

Supplement: Supplementary 1 — Supplementary Figure 1: after subcutaneously injecting in both flanks the HT-29 cells (sh-NC or sh-circIFT80), the mouse body weight changes in the sh-NC, and sh-circIFT80 groups were measured and recorded per week. [file 8081246.f1.png]
