# Supplementary material for: circIFT80 Functions as a ceRNA for miR-142, miR-568, and miR-634 and Promotes the Progression of Colorectal Cancer by Targeting β-Catenin
Source: Dis Markers. 2022 Jun 23;2022:8081246. doi: 10.1155/2022/8081246 (PMC9247842; doi:10.1155/2022/8081246)
Supplement: Supplementary 2 — Table 1: the clinical information of the patients participating in the experiment. Table 2: primer sequences used in quantitative RT-PCR. Table 3: biotin-labeled miRNA probes. [file 8081246.f2.pdf]

Table 1. Clinical date of CRC patients

Number	Gender	Age	T	N	M	TNM
1	Male	79	T2	N0	M0	Grade Ⅰ
2	Male	56	T1	N0	M0	Grade Ⅰ
3	Female	86	T3	N0	M0	Grade Ⅱ A
4	Male	55	T2	N0	M0	Grade Ⅰ
5	Male	66	T2	N1	M0	Grade ⅢA
6	Male	71	T3	N2b	M0	Grade ⅢC
7	Male	63	T4a	N0	M0	Grade Ⅱ B
8	Female	57	T3	N0	M0	Grade Ⅱ A
9	Male	51	T1	N1	M0	Grade ⅢA
10	Male	64	T3	N0	M0	Grade Ⅱ A
11	Female	58	T3	N1	M0	Grade ⅢB
12	Female	66	T3	N0	M0	Grade Ⅱ A
13	Male	61	T3	N1b	M0	Grade ⅢB
14	Female	53	T3	N0	M0	Grade Ⅱ A
15	Male	55	T4a	N0	M0	Grade Ⅱ C
16	Female	75	T4a	N0	M0	Grade Ⅱ C
17	Male	54	T4a	N1	M0	Grade ⅢB
18	Female	56	T4a	N2a	M0	Grade ⅢC

Table 2. Primer sequences used in Quantitative RT-PCR

miRNAs	sequence
hsa-miR-142 F	CATAAAGTAGAAAGCACTACT
hsa-miR-142 R	GTGCGTGTCGTGGAGTCG
hsa-miR-568 F	ATGTATAAATGTATACACAC
hsa-miR-568 R	GTGCGTGTCGTGGAGTCG
hsa-miR-634 F	AACCAGCACCCCAACTTTGGAC
hsa-miR-634 R	GTGCGTGTCGTGGAGTCG
U6 F	CTCGCTTCGGCAGCACA
U6 R	AACGCTTCACGAATTTGCGT

Table 3. Biotin-labeled miRNA probes

miRNAs	sequence
hsa-miR-142 probe	5'-CAUAAAGUAGAAAGCACUACU-biotin-3'
hsa-miR-142 control probe	5'-TCATCACGAAAGATGAAATAC-biotin-3'
hsa-miR-568 probe	5'-AUGUAUAAAUGUAUACACAC-biotin-3'
hsa-miR-568 control probe	5'-CACACATATGTAAATATGTA-biotin-3'
hsa-miR-634 probe	5'-AACCAGCACCCCAACUUUGGAC-biotin-3'
hsa-miR-634 control probe	5'-CAGGTTTCAACCCCACGACCAA-biotin-3'
